# Extraordinary Case of Blood Poisoning

**Published:** 1887-05

**Authors:** F. A. Schmitt

**Affiliations:** Schulenburg, Texas


### EXTRAORDINARY CASE OF BLOOD POISONING. ✓

*By F. A. Schmitt, Schulenburg, Texas.*

For Daniel's Texas Medical Journal.

ON Saturday morning April 2, I was invited by my professional brother, Dr. W. W. Walker, to examine one of his office patients. It was a child 12 months old, whom its parents had carried there for the purpose of calling the Doctor's attention to a peculiar discoloration of part of left leg in the region of the hip. But shortly before had the child's mother noticed the darkening of the skin.

When I first saw the case, one side of the upper part of leg—about the size of an adult's hand—had become perfectly black; extravasation of blood had taken place to such an extent and degree, that the first impression of it, was, that the child had received a severe injury; a blow could not have ruptured the cutaneous vessels so extensively and totally. While the child was at the office of Dr. Walker—perhaps half an hour—discoloration of skin of part of the other leg took place, at first resembling hypostasis.

No constitutional symptoms indicative of a malady of a serious or grave nature being noticeable, and the child, a lively, bright, well-nourished infant, playing on the carpet at my feet, without any inflammatory swelling or pain at or near the site of abnormity, I looked upon the case as certainly one of more than ordinary interest, but by no means as one involving danger or imperilling the child's life within a day or two and and actually proving fatal in 54 hours.

In privately talking over the case with Dr. Walker, he related, that ten years ago, it had been his fate to attend to a similar case—also an infant—in conjunction with Doctor G. W. Jones, the case proving fatal within 50 or 60 hours. He therefore, with propriety, took a more serious view of the case than I did.

We put the child on " expectant treatment " and awaited further developments. The next day—Sunday—did not materially change the condition of the child—constitutionally or otherwise, but at 4 o'clock, P. M., Monday, Dr. Walker was hurriedly summoned to see the child.

He found it extremely prostrated, with evidently dilated pupils, pulse 120, temperature 100; discoloration spreading downwards on right leg; also found one dark spot on elbow, and one on back, but on primarily affected leg it seemed to have become stationary, and somewhat fading.

The child was immediately put on quinine and tr. ferr. mur., and had the diseased parts annointed with carbolized Ungt. petrolii. I saw the child at 10 o'clock, A. M., the same day,—Monday—it was then in a dying condition. The primarily affected parts, (left hip), were now of almost normal color, but discoloration of right leg extended to ankle joint, and here, encircling the leg was seen a 1½ inches wide ring of gangrenous hue, covered with blisters, exuding a dark fluid,—leaving to the foot below its natural color.

The iron and quinine were continued and the leg from knee downwards, covered with a thick layer of pix liquida—the antiseptic virtues of which we, on former occasions, (in gangreuous erysipelas), had experienced.

The child died 54 hours after the first symptoms were noticed. I since heard that Dr. Buford, of Weimar, Colorado county, has had a similar case with like results, but requiring five days in proving fatal. Dr. Buford's attention was called to the case for "curiosity sake," the patient, also an infant, being well otherwise—while attending to another patient in the same family. A few days afterwards Dr. Buford was summoned to this same child and not a little surprised to find it in a dying condition, a large part of its body—hypostasis like—colored or black.

These cases present many points of interest, but as this report has already taken up more space than I had anticipated, I will endeavor briefly to consider some of them, and in the first place the one regarding the exciting cause, (etiology). The child's system had, to all appearances, been the recipient of poison from the outside world, although the parents do not know or recollect of any bite or injury the child to have sustained.

But the child—although in the exclusive care of its attentive mother, may have been approached by a very minute insect and bitten without producing much pain; or, the insect may have been hidden within the wrapper (diaper), and have inoculated its virus—peculiar to itself or being the carrier from the outside world thereof—into abrasions of the skin so often met with among infants. I imagine the materia morbosa to have been some animal poison—the monades of Hueter perhaps spoken of in a former paper (C. R. M. Vol. II., p. 14), which at first but few in number, inducing local traumatism, and with this the peculiar extravasation of the cutis initiating this form of infection. They are then reproduced and, multiplying with rapidity, enter the circulation and after becoming sufficiently numerous decompose the blood or otherwise change the condition of the life giving fluid, as in the above mentioned paper has been explained.

This indeed seems to have been the modus procedendi in the described case as well as the other cases referred to, for in all three of them, time, about 48 hours, was required to produce visible constitutional disturbance, and from then on changing the conditions of nutrition rapidly until disolution ended the process.

As these cases are rare, and certainly interesting, I report them, hoping that other practitioners' experience and wisdom in the premises may lead to more thorough investigation.

